# A NAC Transcription Factor from ‘Sea Rice 86′ Enhances Salt Tolerance by Promoting Hydrogen Sulfide Production in Rice Seedlings

**DOI:** 10.3390/ijms23126435

**Published:** 2022-06-09

**Authors:** Yan Sun, Kaiqiang Song, Miaomiao Guo, Hao Wu, Xuan Ji, Lixia Hou, Xin Liu, Songchong Lu

**Affiliations:** College of Life Sciences, Qingdao Agricultural University, Qingdao 266109, China; sunyan1115@126.com (Y.S.); m17865162636@163.com (K.S.); 17852028923@163.com (M.G.); 13182122109@163.com (H.W.); 13853236381@163.com (X.J.); houlixia78@163.com (L.H.)

**Keywords:** rice, H_2_S, Sea Rice 86, NAC transcription factor, salt tolerance

## Abstract

Soil salinity severely threatens plant growth and crop performance. Hydrogen sulfide (H_2_S), a plant signal molecule, has been implicated in the regulation of plant responses to salinity stress. However, it is unclear how the transcriptional network regulates H_2_S biosynthesis during salt stress response. In this study, we identify a rice NAC (NAM, ATAF and CUC) transcription factor, OsNAC35-like (OsNACL35), from a salt-tolerant cultivar ‘Sea Rice 86′ (SR86) and further show that it may have improved salt tolerance via enhanced H_2_S production. The expression of *OsNACL35* was significantly upregulated by high salinity and hydrogen peroxide (H_2_O_2_). The OsNACL35 protein was localized predominantly in the nucleus and was found to have transactivation activity in yeast. The overexpression of *OsNACL35* (*OsNACL35-OE*) in japonica cultivar Nipponbare ramatically increased resistance to salinity stress, whereas its dominant-negative constructs (SUPERMAN repression domain, SRDX) conferred hypersensitivity to salt stress in the transgenic lines at the vegetative stage. Moreover, the quantitative real-time PCR analysis showed that many stress-associated genes were differentially expressed in the *OsNACL35-OE* and *OsNACL35-SRDX* lines. Interestingly, the ectopic expression of *OsNACL35* triggered a sharp increase in H_2_S content by upregulating the expression of a H_2_S biosynthetic gene, Os*DCD1*, upon salinity stress. Furthermore, the dual luciferase and yeast one-hybrid assays indicated that OsNACL35 directly upregulated the expression of *OsDCD1* by binding to the promoter sequence of *OsDCD1*. Taken together, our observations illustrate that OsNACL35 acts as a positive regulator that links H_2_S production to salt stress tolerance, which may hold promising utility in breeding salt-tolerant rice cultivar.

## 1. Introduction

In the era of climate change, soil salinity has become a severe menace to plant growth and development and agricultural productivity all over the world. Soil salinity imposes both osmotic and ionic stresses on plant cells, causing oxidative stress and ionic toxicity, ultimately leading to growth retardation and decreased agricultural yield [1,2,3,4,5]. To counteract the adverse effect of salinity stress, plants have evolved a myriad of genetic mechanisms to alter physiological and developmental responses [6]. Upon exposure to salt stress, a set of stress-induced genes are activated to orchestrate salt tolerance responses in plants. Rice, one of the most important cereal crops, provides food for half of the global human population and is highly susceptible to salt stress [2,4]. A salt-tolerant rice cultivar, “Sea Rice 86” (SR86), can survive salinity conditions equivalent to 1/3 concentration of sea water. The SR86 rice is thus an ideal candidate plant for identifying salt-stress-related genes and revealing stress-response pathways [7]. The identification and characterization of key salt-stress-tolerance genes from SR86 and further understanding of the regulatory mechanisms of rice salt stress responses have important implications for improving salt tolerance in crops and global food security.

High salinity induces changes in the expression of many stress-related genes [8]. Among these, various families of transcription factors (TFs) (e.g., NAC, AP2/ERF, MYB, WRKY, bZIP, bHLH and CAMTA) have been identified and characterized [9,10,11,12,13,14,15]. The NAC TFs comprise one of the largest families of plant-specific TFs with 117 and 151 predicted members in *Arabidopsis* and rice [16], respectively. The NAC proteins feature a highly conserved N-terminal DNA-binding domain and a variable C-terminal transcription activation domain [17]. Increasing evidence supports the functional significance of NAC TFs in controlling many aspects of plant development, such as root development [18,19,20], leaf senescence [10,21], floral development [22], and seed germination [18,23]. Studies have also shed light on their roles in plant responses to environmental stress conditions [5,24,25,26], including salt stress [2,5,24]. Some reports show that transgenic rice lines overexpressing stress-related NAC TF genes exhibit improved salt resistance without yield penalty, implying that these NAC TFs have a potential application in breeding salt-resistant rice and possibly other cereals [26,27].

Although it has been well-established that plants synthesize and release hydrogen sulfide (H_2_S) [28], the cellular and physiological functions of H_2_S in plants have been recognized only recently [29,30]. Studies have shown that L-cysteine desulfhydrase (LCD), D- cysteine desulfhydrase (DCD) and DES1 (O-acetyl-L-serine (thiol) lyase (OASTL) family) are essential enzymes degrading L-Cys and D-Cys into H_2_S in plants [30]. As a “gasotransmitter”, H_2_S can affect a number of developmental and physiological processes in plants, such as seed germination, root development, stomatal movement, fruit ripening, leaf senescence, and plant responses to abiotic stresses [31,32,33,34,35,36,37,38,39]. Concerning the mechanism of action for H_2_S, recent reports have shown that H_2_S can regulate multiple signaling pathways through the persulfidation of target proteins [40,41,42,43,44]. For instance, H_2_S was found to modulate ABA signaling by the persulfidation of SnRK2.6 and ABI4, key components of the ABA signaling pathway, resulting in changes in their enzymatic activity. In the context of salinity response, studies show that the exogenous application of NaHS, a H_2_S donor, enhances plant salt tolerance [38,40], implicating H_2_S as a positive signal to promote salt tolerance.

Despite findings on some NAC TFs and H_2_S in salinity responses [2,5,24,45], the relationship between the two remains unknown. In our study of salt tolerance of sea rice SR86, we identified a NAC TF, *OsNACL35,* as an effector of salt tolerance and have linked this transcriptional factor to the biosynthesis of H_2_S. We thus propose that OsNACL35 is a positive regulator of salt stress tolerance by promoting H_2_S production.

## 2. Results

### 2.1. Isolation and Sequence Analysis of OsNACL35

To isolate salt stress-associated genes from SR86, we performed a transcriptome analysis using the total RNA from seedlings grown under salt-stress conditions. In this study, we focused on *OsNACL35* for further investigation.

**Figure 1:**
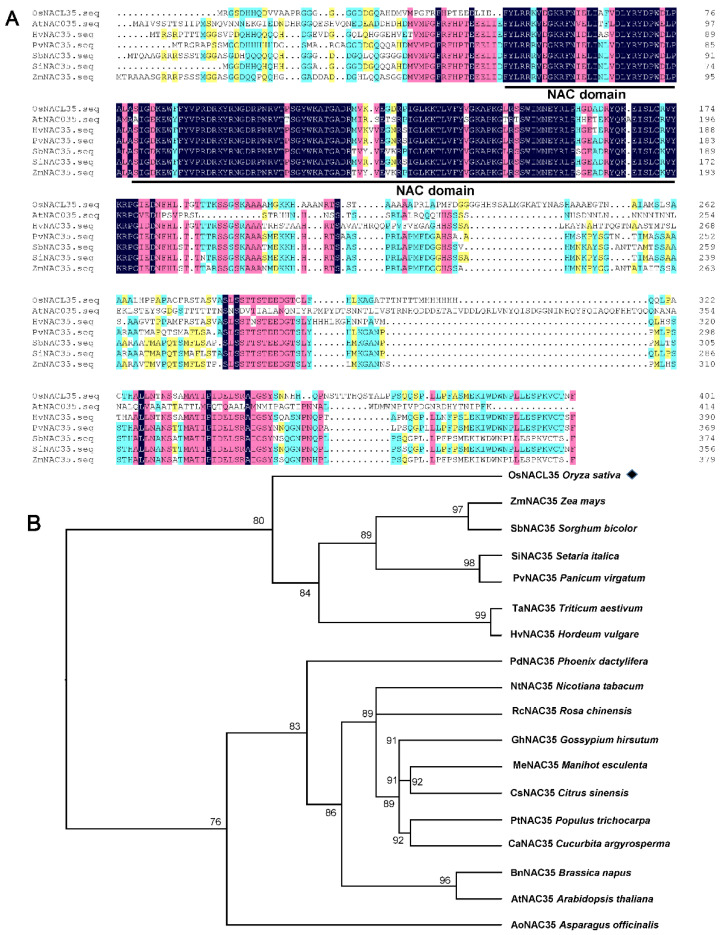
Phylogenetic analysis of OsNACL35. (**A**) Multiple protein sequence alignment of OsNACL35 with other NAC35-like proteins from Arabidopsis, *Hordeum, Panicum, Sorghum, Setaria* and *maize*. The conserved NAC domains are marked as bold line. (**B**) Phylogenetic tree analysis of OsNACL35 (indicated by black diamond) with other known plant NAC35 proteins. The accession numbers of these NAC35 protein as follows: *Zea mays* (NP_001159214.1), *Sorghum bicolor* (XP_002443756.1), *Setaria italica* (XP_004973013.1), *Panicum virgatum* (XP_039852575.1), *Triticum aestivum* (XP_044436564.1), *Hordeum vulgare *(XP_044958989.1), *Phoenix dactylifera* (XP_008804076.1), *Nicotiana tabacum* (XP_016509171.1), *Rosa chinensis* (XP_024172915.1), *Gossypium hirsutum* (XP_016678255.1), *Manihot esculenta* (XP_021615085.1), *Citrus sinensis* (XP_006470800.1), *Populus trichocarpa* (XP_024452941.1), *Cucurbita argyrosperma* (KAG7031334.1), *Brassica napus* (XP_013713097.1), *Arabidopsis thaliana* (AT2G02450.1), and *Asparagus officinalis* (XP_020252109.1). Multiple alignments and phylogenetic tree of OsNACL35 and its homologs proteins were performed through the software DNAMAN v.9.0.

The full-length CDS sequence of OsNACL35 encodes a protein with 402 amino acid residues (Figure 1A). the OsNACL35 protein was predicted to contain a conserved NAC domain in the N-terminal region through SMART analysis (Figure 1A). We constructed a phylogenetic tree using OsNACL35 and homology with NAC orthologs in other higher plant species. As expected, OsNACL35 showed the highest sequence identity to various NAC35 TFs from other monocotyledonous crops (Figure 1B).

### 2.2. OsNACL35 Is Localized in the Nucleus and Displays Transactivation Activity in Yeast

To examine the subcellular localization of the OsNACL35 protein, we built a construct containing a cauliflower mosaic virus (CaMV) 35S promoter driving the expression of the OsNACL35–GFP fusion. The construct and the nuclear marker Nu-mCherry was co-transformed into leaves of N. *benthamiana* through the agrobacterium-mediated method. The fluorescence signals of mCherry and GFP completely overlapped, indicating that the OsNACL35–GFP fusion protein was localized in the nucleus of the cells.

**Figure 2:**
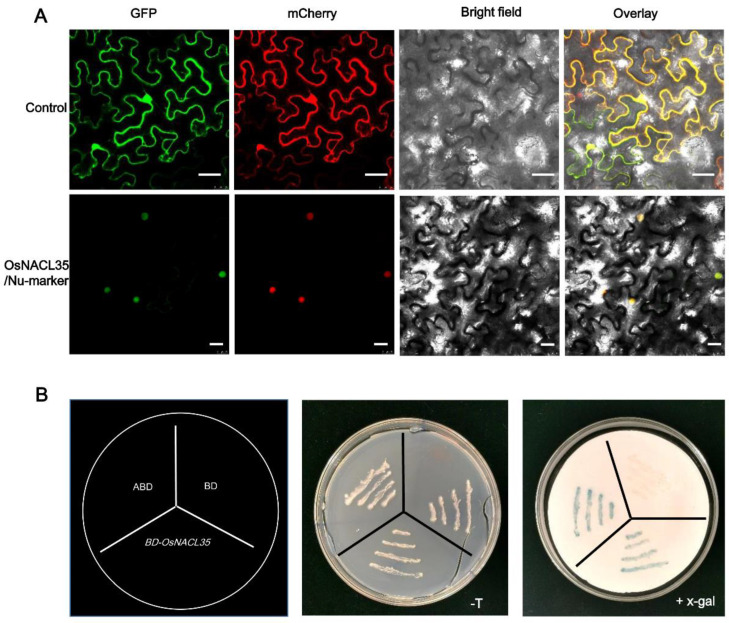
Subcellular localization and transactivation analysis of OsNACL35. (**A**) OsNACL35 is localized in nucleus. 35S:GFP, 35S:OsNACL35-GFP, and nuclear marker (BES1n-mCherry) were expressed in the leaves of *Nicotiana benthamiana*. Leaves were collected at 48 h after infiltration for observation under a confocal laser scanning microscope (Bar = 50μm). (**B**) Transactivation assay of OsNACL35 in the yeast strain AH109. Recombinant constructs of pGBKT7–OsNACL35 were expressed in the yeast strain AH109. The vector pGBKT7 was expressed in yeast as a control, and ABD as a positive control. The plates were incubated for 3 days and then subjected to the galactosidase assay.

We further tested if OsNACL35 acted as a transcriptional activator in the yeast system. Using *pGBKT7-AD* as the positive control and the empty vector pGBKT7 as the negative control, we transformed the *pGBKT7–OsNACL35* construct into the AHA109 yeast strain. All yeast transformants grew on the SD/-Trp plates, suggesting the presence of the vector plasmids (Figure 2B). In addition, the yeast cells carrying *pGBKT7–OsNACL35* and positive constructs showed β-galactosidase activity (Figure 2B), indicating the expression of the beta-gal reporter gene as a result of OsNACL35 being a transactivation factor.

### 2.3. Expression Pattern of OsNACL35 in SR86

**Figure 3:**
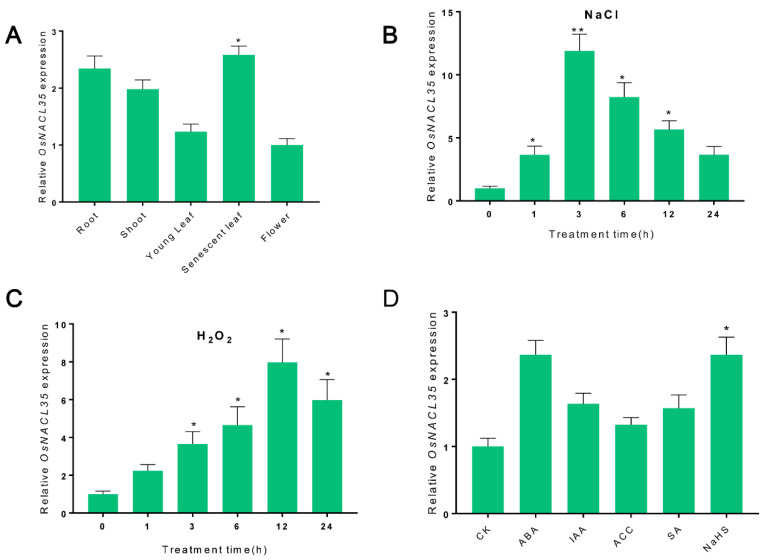
Expression patterns of *OsNACL35* in SR86. (**A**) Expression profiles of *OsNACL35* in different tissues of SR86. Data were normalized against the flower sample. (**B**,**C**) Time course of the OsNACL35 transcript level under salt stress and H2O2 treatment. Relative expression was calculated against the expression level at 0 h. (**D**) Expression profiles of *OsNACL35* upon different hormone and NaHS treatments. The expression level in CK was calculated as reference. The values are means ±SD of three biological replicates. Asterisks indicate statistically significant differences (* *p* < 0.05, ** *p* < 0.01) from the control check (CK).

The expression profile of *OsNACL35* in SR86 was monitored by quantitative real-time PCR (qRT-PCR). To further study the temporal and spatial expression pattern of *OsNACL35* in SR86, various tissues (root, shoot, young leaf, senescent leaf, and flower) of SR86 were harvested, and the tissue distribution of the *OsNACL35* transcript was detected by qRT-PCR. The expression analysis showed that *OsNACL35* was expressed in all the tissues described above, with the highest level of expression in the senescent leaf, followed by root and shoot, and the lowest level of expression in the flower (Figure 3A).

Under salt-stress conditions, the expression level of *OsNACL35* started to accumulate after 1 h of exposure to salt stress (200 mM NaCl), and peaked after 3 h of salt-stress treatment, showing a nine-fold increase over that in the control check (CK) plants (Figure 3B), and then declined gradually. When plants were exposed to H_2_O_2_ treatment, a similar increase in the *OsNACL35* transcript was observed (Figure 3C). Moreover, the significant induction of the *OsNACL35* gene was monitored upon exposure to the ABA and NaHS (H_2_S donor) treatments. The expression level of *OsNACL35* showed no obvious increase upon exposure to the exogenous application of IAA, ACC, and SA (Figure 3D).

### 2.4. Overexpression of OsNACL35 Confers Tolerance to Salt Stress

**Figure 4:**
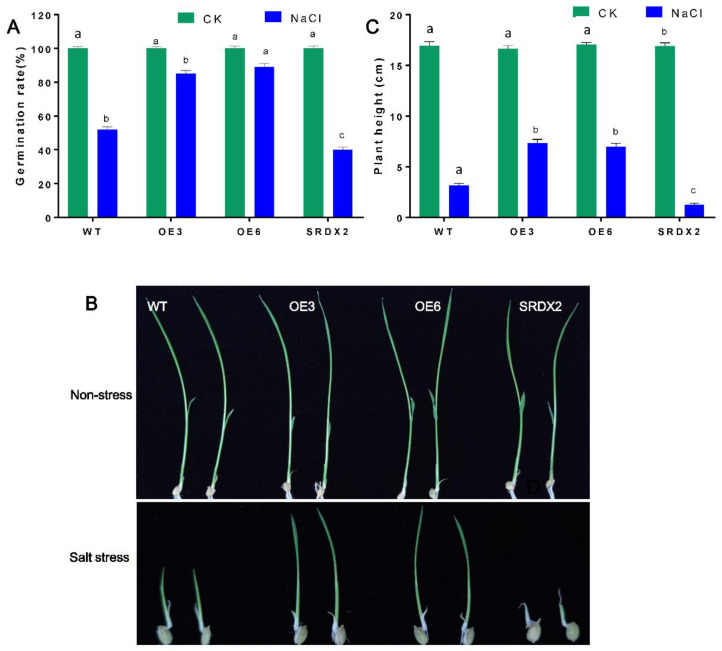
Responses of seed germination and seedling growth to NaCl treatment. (**A**) Germination rates: seeds of transgenic lines and WT were soaked in distilled water for 1 day and then scattered on a 1/2 MS medium with 100 mM NaCl for 5 days. (**B**) Phenotypes: seeds were placed in distilled water for 2 days, and then transferred to a 1/2 MS solution containing 150 mM NaCl for 14 days. Mean and SD are shown (n = 20). (**C**) Shoot height of the seedlings in (**B**). Statistical analyses were performed by a two-way ANOVA, followed by a Tukey’s multiple comparison test. Different letters indicate significant differences at *p* < 0.05.

To decipher the biological function of OsNACL35, we generated *35S:OsNACL35* overexpression constructs and the dominant-negative vectors *35S:OsNACL35-SRDX*. Subsequently, these recombinant constructs were transformed into rice (*Oryza sativa* cv. Niponbare) through the agrobacterium-mediated method. We obtained 10 lines of *OsNACL35*-overexpressing plants (*OsNACL35-OE*), and 5 lines of *OsNACL35-SRDX* transgenic plants (SRDX). Two independent homozygous T3 overexpressing lines (OE3 and OE6) and one SRDX (SRDX2) line, which grew normally with no stunting, were selected for further study (Figure 4A).

To check the tolerance of transgenic rice plants to salt stress, the transgenic plants and the wild-type plants (WT) were subjected to salt stress. Under normal conditions, these transgenic plants displayed a similar phenotype to WT. However, the *OsNACL35-OE* exhibited faster germination than those of WT with exposure to 150 mM NaCl treatment, while the germination rate of *SRDX* lines were the lowest. Moreover, 2-day-old seedlings were subjected to 150 mM NaCl treatment for 10 days, and shoot height was measured. There was no difference between transgenic and WT plants under normal conditions in a hydroponic solution (Figure 4B). Salt stress caused the smallest reduction in the plant height of *OsNACL35-OE* compared to that of WT, yet *SRDX2* plants displayed the greatest reduction in plant height (Figure 4B,C).

**Figure 5:**
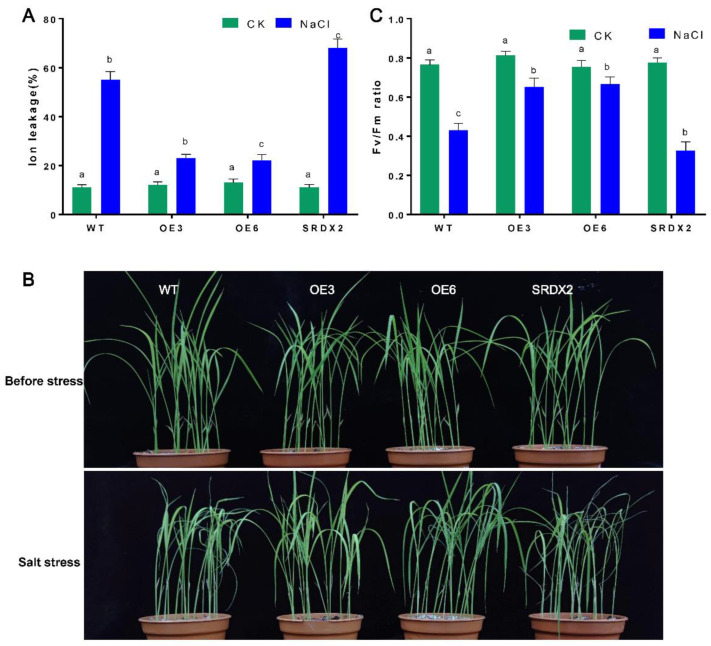
Effect of salt stress on WT and transgenic rice plants. (**A**) Ion leakage assay of these plants upon salt stress. Mean and SD are shown (n = 20) (**B**) Phenotypes of WT and transgenic rice plants grown in soil under normal conditions and salt stress (350 mM). Independent experiments were repeated three times. (**C**) Fv/Fm of rice plants. The 21-day-old rice seedlings grown under the control and salt-stress (200 mM NaCl) conditions for 4 days. Mean and SD are shown (n = 20). Statistical analyses were performed by a two-way ANOVA, followed by a Tukey’s multiple comparison test. Different letters indicate significant differences at *p* < 0.05.

To evaluate how *OsNACL35-OE* would perform in soil conditions, the transgenic lines and WT plants were grown on 1/2 MS plates for 4 days; the seedlings then were transferred into soil and grown under normal conditions for another 3 weeks. During these 3 weeks, these plants were indistinguishable (Figure 5). When exposed to a 300 mM NaCl treatment for 5 days, *OsNACL35-OE* performed better than WT, and exhibited alleviated symptoms of salinity damage (e.g., wilting and leaf-rolling) (Figure 5B). Accumulating evidence demonstrates that electrolyte leakage could reflect the stress-induced damage of plasma membrane. Therefore, the rates of electrolyte leakage of these plants were detected upon exposure to 300 mM NaCl. The results indicate that no obvious difference was observed between these transgenic lines and WT plants under non-stress conditions. However, the electrolyte leakage rates of *OsNACL35-OE* displayed lower values than those of WT under high-salt-stress conditions, yet a higher rate of electrolyte leakage was observed in *SRDX2* plants (Figure 5A). In addition, we also measured the Fv/Fm values that were an indicator of the photochemical efficiency of photosystem II (PSII). Upon exposure to salt stress, the Fv/Fm ratio was significantly increased in OE3 and OE6 plants compared with that in the WT plants, and it was significantly lower in *SRDX2* than in WT (Figure 5B). These results suggest that OsNACL35 could positively regulate salt tolerance in rice.

### 2.5. Overexpression of OsNACL35 Promotes Scavenging of Reactive Oxygen Species and Accumulation of Osmotic Substance and H_2_S

**Figure 6:**
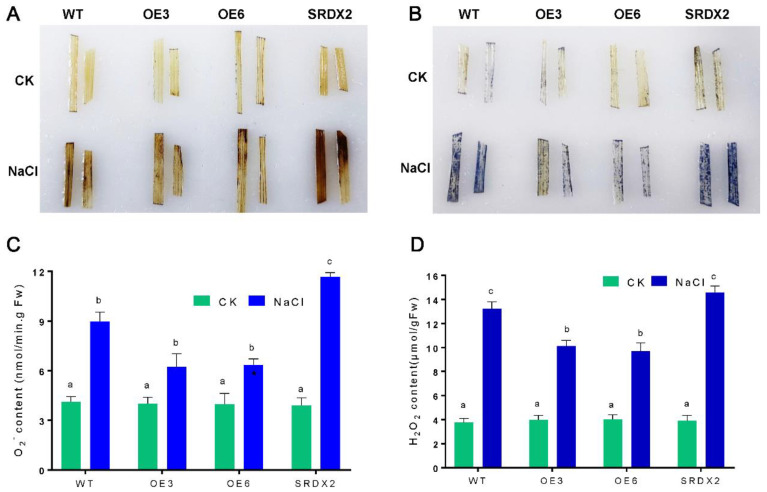
OsNACL35 modulates ROS content under salt stress. (**A**) DAB staining. (**B**) NBT staining. Three independent experiments were conducted (n ≥ 15), showing similar results. (**C**) O^2−^ content. (**D**) H_2_O_2_ content. The 21-day-old rice seedlings grown under control and salt-stress (350 mM NaCl) conditions for 4 days. Statistical analyses were performed by a two-way ANOVA, followed by a Tukey’s multiple comparison test. Different letters indicate significant differences at *p* < 0.05.

**Figure 7:**
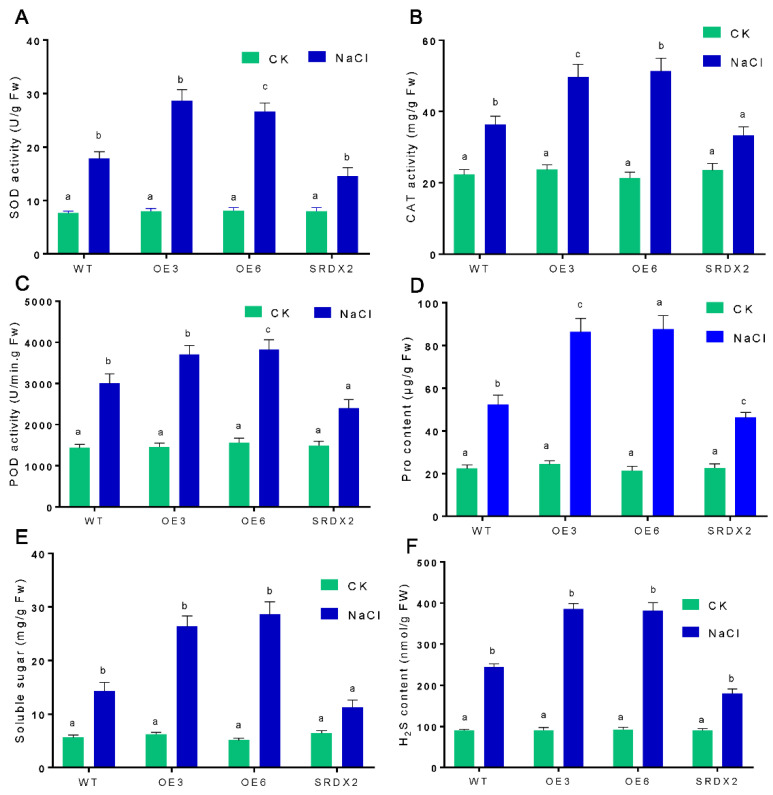
Effect of salt stress on antioxidant enzymes (**A**–**C**) and contents of osmoprotectant (**D**,**E**) and H_2_S (**F**) in the wild-type and transgenic rice plants. The 21-day-old rice seedlings grown under control and salt-stress (350 mM NaCl) conditions for 4 days. Statistical analyses were performed by a two-way ANOVA, followed by a Tukey’s multiple comparison test. Different letters indicate significant differences at *p* < 0.05.

To further reveal the physiological function of OsNACL35 in salt stress, we measured the reactive oxygen species (ROS) accumulation via the DAB and NBT staining, and the absorbance spectrophotometry method. The results show that *OsNACL35-OE* plants had a lower level of H_2_O_2_ and O_2_^−^ than WT, while *SRDX2* lines accumulated obviously more ROS upon exposure to high-salinity stress (Figure 6A–D). Furthermore, we detected the activities of the major antioxidant enzymes. Under normal conditions, the activities of superoxide dismutase (SOD), peroxidase (POD), and catalase (CAT) were indistinguishable among *OsNACL35-OE*, WT, and *SRDX* plants. When exposed to salt stress, there were sharp increases in the activities of SOD, POD, and CAT in the rice seedlings. Especially, the activities of these antioxidant enzymes were significantly higher in *OsNACL35-OE*, but lower in the *SRDX* lines (Figure 7A–C). Moreover, we compared the accumulation of osmotic substance (e.g., proline and soluble sugar) of WT, *OsNACL35-OE*, and *SRDX* plants under salt-stress conditions. Upon exposure to salt stress, the *OsNACL35-OE* lines accumulated greater amounts of proline and soluble sugar compared to WT, while the concentration of proline and soluble sugar was lower in *SRDX2* (Figure 7D,E). According to the NaHS-induction of *OsNACL35* described above, we tested the endogenous hydrogen sulfide production of WT, *OsNACL35-OE*, and *SRDX* plants under salt-stress conditions. As shown in Figure 7F, under salinity stress, the *OsNACL35-OE* lines accumulated more H_2_S than WT, whereas H_2_S production was much lower in *SRDX2*. These results indicate that OsNACL35 modulates ROS scavenging and accumulation of osmotic substance and H_2_S.

### 2.6. Overexpression of OsNACL35 Alters the Expression of Various Stress-Related Genes

**Figure 8:**
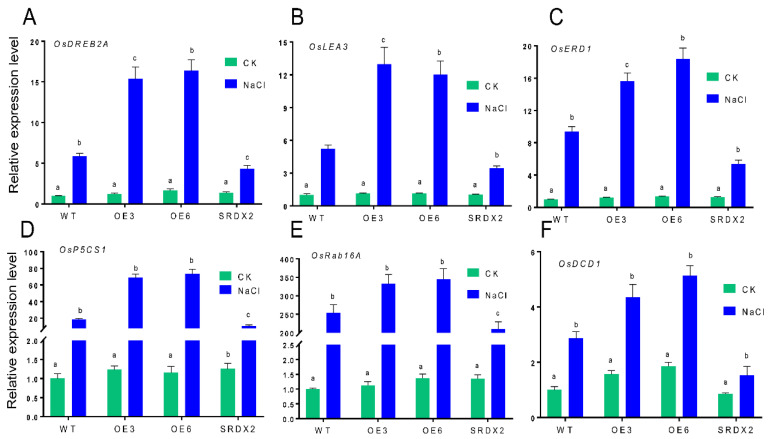
Expression levels of some salt-responsive genes and hydrogen sulfide synthesis gene in wild-type and transgenic plants. (**A**) *OsDREB2A*. (**B**) *OsLEA3*. (**C**) *OsERD1.* (**D**) *OsP5CS1.* (**E**) *OsRab16A.* (**F**) *OsDCD1*. Total RNA was extracted from 21-day-old rice seedlings grown under control and salt-stress (350 mM NaCl) conditions for 4 days. Statistical analyses were performed by a two-way ANOVA, followed by a Tukey’s multiple comparison test. Different letters indicate significant differences at *p* < 0.05.

To confirm the molecular mechanism of *OsNACL35* involved in salt stress, we examined the expression level of a myriad salt stress-related genes from previous reports, such as *OsDREB2A*, *OsLEA3*, *OsERD1*, *OsP5CS1,* and *OsRab16A* [6,44]. The transcript levels of these salt-stress-induced genes were further determined by qRT-PCR. Under normal conditions, these transgenic and WT plants had no obvious difference in transcript levels of genes mentioned above. However, significant increases in the expression of *OsDREB2A, OsLEA3*, *OsERD1*, *OsP5CS1*, and *OsRab16A* were observed in *OsNACL35-OE*, while the transcript levels of those genes were obviously down-regulated in *SRDX2* lines compared with WT (Figure 8A–E). Interestingly, it was found that the transcript level of *OsDCD1*, a novel H_2_S biosynthesis gene, was remarkably up-regulated in *OsNACL35-OE* plants under salt stress, compared with WT (Figure 8F). The above results demonstrated that OsNACL35 could play a crucial role, direct and/or indirect, in regulating the expression of those salt-stress-related genes and H_2_S synthesis genes during plant responses to salt stress.

### 2.7. OsNACL35 Directly Regulates the Expression of OsDCD1 and OsLEA3

**Figure 9:**
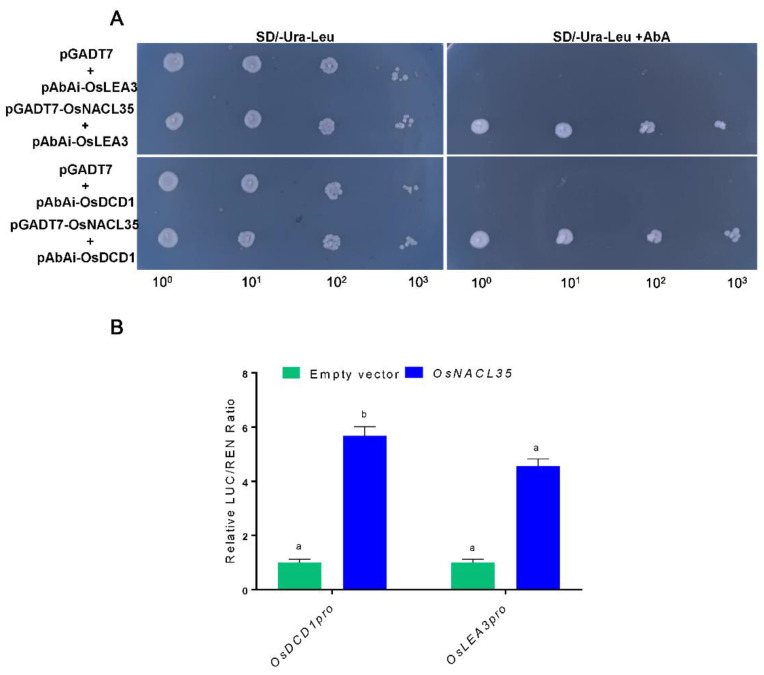
*OsDCD1*and *OsLEA3* are the direct target genes of OsNAL35. (**A**) Interaction of OsNACL35 with the promoters *of OsDCD1* and *OsLEA3* through yeast one-hybrid assays. The yeast cells were grown on SD/-Leu/-Ura/+AbA medium. (**B**) Interaction of OsNACL35 with the promoters of *OsDCD1* and *OsLEA3* by dual-luciferase reporter activation assays in tobacco. Three independent experiments were conducted, showing similar results. Statistical analyses were performed by a two-way ANOVA, followed by a Tukey’s multiple comparison test. Different letters indicate significant differences at *p* < 0.05.

To deeply investigate the molecular mechanism of OsNACL35 modulating plant responses to salt stress, we checked whether OsNACL35 could directly regulate those genes, such as *OsDREB2A*, *OsLEA3*, *OsERD1*, *OsP5CS1*, *OsRab16A*, and *OsDCD1.* Yeast one-hybrid assay was performed to examine whether OsNACL35 could bind to the promoters of these genes. We generated *pGADT7–OsNACL35* and *pAbAi* recombinant plasmids containing the promoter fragments of the above genes. The yeast one-hybrid assay exhibited that OsNACL35 was only directly bound to the promoter sequences of *OsLEA3* and *OsDCD1* (Figure 9A). Furthermore, we performed the dual-luciferase assay in the *N. benthamiana* system to test the interaction mentioned above. The promoter fragments of *OsLEA3* and *OsDCD1* were ligated into *pGreenII0800-LUC*, and the ORF of *OsNACL35* was fused with *pGreenII62-SK*. The dual-luciferase reporter system showed that the LUC/REN ratio was higher than that of the control, when *pGreenII62-SK-OsNACL35* was co-infiltrated with *pGreenII0800-LUC-OsDCD1pro* or *pGreenII0800-LUC-OsLEA3pro* (Figure 9B). Overall, these results indicate that OsNACL35 could be directly bound to the promoters of *OsLEA3* and *OsDCD1*, thereby improving plant resistance to salt stress.

### 2.8. H_2_S Acts a Positive Molecule to Promote Salt Stress Tolerance in Rice Seedlings

**Figure 10:**
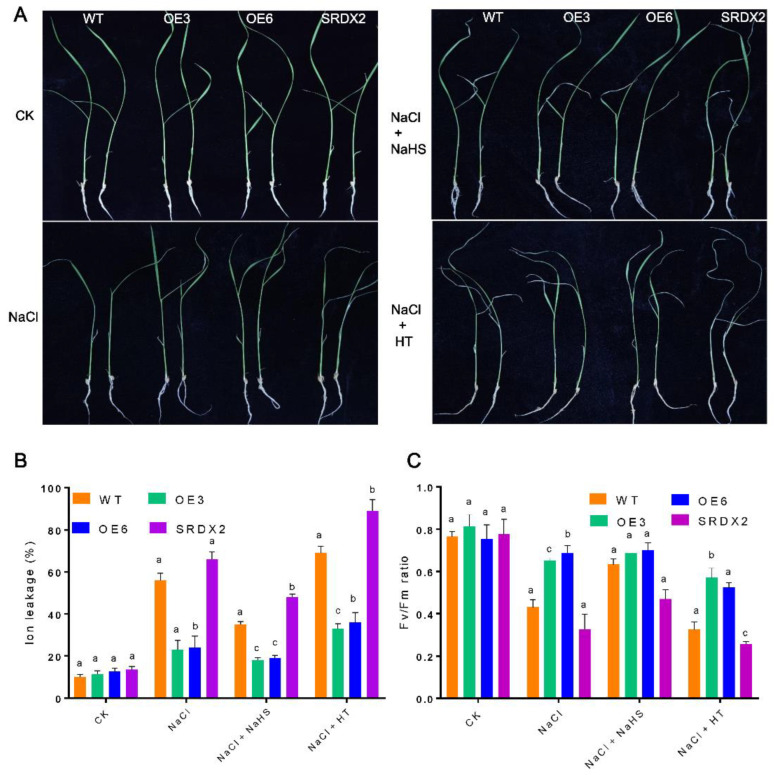
Effects of NaHS and hypotaurine (HT) on salt tolerance in WT and transgenic lines. The 14-day-old seedlings were pretreated with or without NaHS (100 μM) and HT (2 mM) for 6 h, and then shifted to a 1/2 Murashige and Skoog (MS) solution with or without NaCl (200 mM) for another 48 h. (**A**) Phenotypes: ion leakage (**B**) and Fv/Fm (**C**) were measured. Seedlings without chemical treatment were regarded as the control check (CK). Values are means ± SE of three independent experiments with at least three replicates for each. Three independent experiments were conducted, showing similar results. Statistical analyses were performed by a two-way ANOVA, followed by a Tukey’s multiple comparison test. Different letters indicate significant differences at *p* < 0.05.

**Figure 11:**
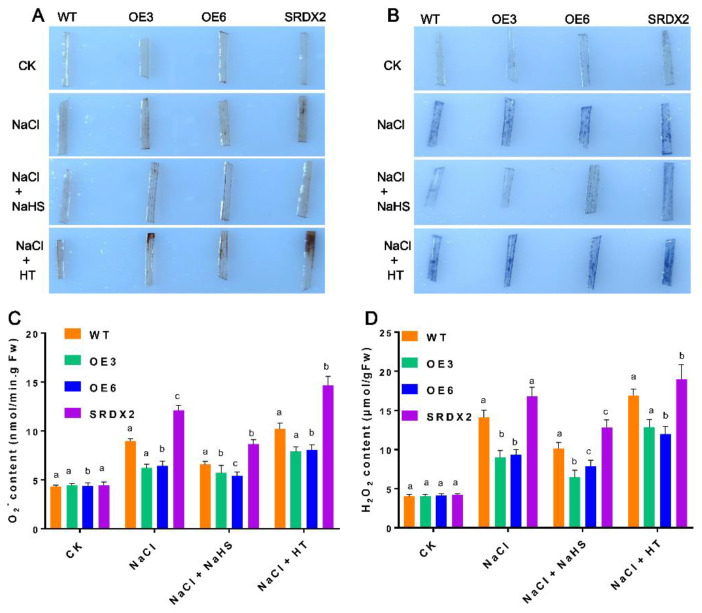
Effects of NaHS and hypotaurine (HT) on ROS content in WT and transgenic lines under salt stress. (**A**) DAB staining. (**B**) NBT staining. (**C**) O_2_^−^ content. (**D**) H_2_O_2_ content. The 14-day-old seedlings were pretreated with or without NaHS (100 μM) and HT (2 mM) for 6 h, and then shifted to a 1/2 Murashige and Skoog (MS) solution with or without NaCl (200 mM) for another 48 h. Seedlings without chemical treatment were regarded as the control check (CK). Values are means ± SE of three independent experiments with at least three replicates for each. Statistical analyses were performed by a two-way ANOVA, followed by a Tukey’s multiple comparison test. Different letters indicate significant differences at *p* < 0.05.

To evaluate whether H_2_S acts downstream of OsNACL35 to mediate the salt signaling pathway in rice plants, we performed pharmacological experiments using sodium hydrosulfide (NaHS, a H_2_S donor) and hypotaurine (HT, a H_2_S scavenger). We compared growth status, electrolyte leakage, ROS content and Fv/Fm of WT, *OsNACL35-OE,* and *SRDX* lines under various treatment conditions. After three days of a 150 mM NaCl treatment in a hydroponic medium, the pretreatment with NaHS significantly alleviated growth inhibition caused by salt stress, while the pretreatment with HT remarkably aggravated the symptoms of salinity damage compared with those seedlings treated with NaCl alone (Figure 10A). We measured the electrolyte leakage of these plants upon different treatments. The results of electrolyte leakage analysis showed that the pretreatment with NaHS decreased the electrolyte leakage of these plants, yet HT dramatically increased electrolyte leakage (Figure 10B). The Fv/Fm values showed similar effects of NaHS and HT (Figure 10C). Additionally, the results of ROS staining (e.g., DAB and NBT staining) and ROS content also demonstrated that NaHS promoted the scavenging of ROS, while HT aggravated the accumulation of ROS (Figure 11A–D). Based on these results, we conclude that H_2_S could act downstream of OsNACL35 to mitigate the toxic effect of salt stress in rice plants.

## 3. Discussion

A major challenge faced by modern agricultural production is the increasing demand for food, while soil salinization is becoming more and more serious; subsequently, arable land is being rapidly lost [46]. Salt stress is one of the most severe environmental stresses constraining plant growth and development and crop yield [47]. Rice, as one of the staple food crops for more than half of the world’s population, is a salt-sensitive crop. Salinity stress suppresses photosynthesis and growth, leading to biomass loss, as well as partial sterility, which ultimately results in the reduction in rice yield [48]. Therefore, the in-depth exploration of the salt tolerance mechanism of rice and improvement in rice salt tolerance have extraordinary significance for ensuring food security. Compared with cultivated rice, SR86 is much more tolerant to alkaline salt stress. So, it is an ideal candidate plant for isolating salt-stress-related genes and for revealing the salt-stress signaling pathway. In recent years, many studies have reported that members of the NAC transcription factor family play a key role in the resistance to high-salinity stress in rice [49]. The expression of the transcription factor OsNAC5 in rice is induced by abiotic stresses, such as drought, cold, and high salinity [50]. OsNAC2 is highly expressed in rice roots, and its expression peaked 12 h after a treatment of high salinity [51]. In our study, OsNACL35, one of the members of the NAC transcription factor family from SR86 (Figure 1A,B), was abundantly expressed in rice leaves and roots, and its transcript level is up-regulated by high salinity, H_2_O_2_, and NaHS treatments. This strongly implies that OsNACL35 may be involved in responses to salt stress in rice. However, the relationship between OsNACL35 and H_2_S remains largely unknown. At present, a few NAC transcription factors that have been reported to be involved in the process of salt stress in rice play a positive regulatory role in the salt tolerance of rice. For example, SNAC1 could greatly improve drought and high-salt tolerance in rice by reducing the transpiration rate [52]. The study by Hong et al. presented that the stress-responsive NAC transcription factor ONAC022, overexpressed in rice, resulted in increased drought and salt tolerance [23]. In rice, OsNAC45 was induced by high salinity and the knockout mutant exhibited higher levels of salt sensitivity [5]. Another salt-inducible gene is *OsNAC3*, whose overexpression enhanced salt tolerance in rice [53]. In our study, compared to the WT, plants overexpressing *OsNACL35* performed better under salt stress. In addition, the dominant chimeric repressor-mediated suppression of OsNACL35 function in *OsNACL35-SRDX* plants exhibited a significant sensitivity to salinity stress. This suggests that the OsNACL35 identified in this study is characterized as a positive regulator in the mechanism of salt tolerance in rice.

Numerous studies have revealed that ROS scavenging capacity and ion balance are related to plants’ tolerance to salt stress [54,55]. The mechanisms of salt tolerance in plants involve complex stress signaling, including osmoregulation, ion homeostasis, and free radical scavenging [56]. Salt stress causes the excessive accumulation of ROS produced by NADPH oxidase, which can damage DNA, proteins, and carbohydrates in plant cells, and eventually leads to cell death [57]. Moreover, high concentrations of salt, especially sodium chloride (NaCl), in the growing environment of plants can cause osmotic and ionic stresses, resulting in changes in the K^+^/Na^+^ ratio and elevated Na^+^ and Cl^-^ concentrations, resulting in metabolic disorders in plants [58]. In this work, *OsNACL35* overexpression in plants exhibited lower ROS accumulation, higher ROS scavenging enzyme (SOD, POD, and CAT) activities, and a lower MDA concentration in a salt-stressed environment. At the same time, *OsNACL35* overexpression in plants also showed a lower ion leakage rate. This suggests that OsNACL35 may be involved in regulating the expression of key genes in the ROS signaling pathway and ion osmosis mechanism, thereby improving the ability of plants to resist oxidative stress and maintaining ion balance in a salt-stressed environment and enhancing the salt tolerance of rice.

Hydrogen sulfide (H_2_S) is the third gaseous signal molecule that is excavated after nitric oxide and carbon monoxide in animals, and is closely related to body health [59]. Recent studies have shown that, in plant systems, the application of exogenous H_2_S donors can significantly enhance the tolerance of plants to abiotic stresses, such as drought, high salinity, and toxic heavy metals [45,60,61]. More and more attention has been paid to the mechanism by which the signaling molecule H_2_S in the plant system regulates other signaling pathways involved in various abiotic stress responses [62]. It had been reported that H_2_S could improve drought tolerance in rice by re-establishing redox homeostasis and activating the ABA signaling pathway [63], and could also alleviate aluminum toxicity by reducing aluminum content in rice [64]. However, few studies on the role of H_2_S in the response to salt stress in rice have been reported. Mostofa et al. found that endogenous H_2_S content increased in rice treated with 150 mM NaCl [45]. Consistent with previous research results, it was found in our study that the endogenous H_2_S content of rice under salt stress was significantly higher than that of plants under a normal environment, which fully implied that H_2_S played an important role in the response of rice to salt stress. H_2_S has been shown to play a positive regulatory role in inhibiting cadmium toxicity in rice by modulating the physiological and biochemical reactions induced by high concentrations of cadmium [65]. However, the study by Lv et al. showed that endogenous H_2_S synthesis induced by low-concentration cadmium stress (<4 μmol/L) in *Brassica rapa* could trigger changes in the balance of hydrogen peroxide and oxygen radicals, which ultimately inhibited root elongation [66]. These results suggest that H_2_S may play a dual role in plant stress response. In this study, the application of exogenous H_2_S donors could improve the salt tolerance of rice, while the application of H_2_S scavengers increased the salt sensitivity of rice. It can be observed that H_2_S plays a positive regulatory role in rice resistance to salt stress.

Previous studies have shown that a variety of enzymes in plants are involved in the biosynthesis of H_2_S, such as the cysteine desulfhydrases (LCD, DCD1, DCD2 and DES1), the cystine desulfurases (NFS1 and NFS2,), and β-cyanoalanine synthases (CYS-C1, CYS-D1), etc. [30]. The expression or inhibition of these proteins significantly affected the synthesis of H_2_S in plants. Zhang et al. found that the WRKY transcription factor enhanced cadmium tolerance in Arabidopsis by regulating the expression of D-cysteine desulfhydrase (DCD) genes and promoting the synthesis of H_2_S [67]. In this study, it was found that the application of the exogenous H_2_S donor induced the expression of *OsNACL35*, while the H_2_S scavenger aggravated the toxic effect of high salt on *OsNACL35-OE*, suggesting that the synthesis of H_2_S in rice might be regulated by OsNACL35 transcription factors. The H_2_S content was increased in *OsNACL35*-overexpressing lines and decreased in *OsNACL35-SRDX* lines, which further verified our speculations. The expression of the H_2_S-synthesis-related genes *OsDCD1* in *OsNACL35* transgenic lines and the results of a series of molecular biochemical experiments confirmed that OsNACL35 could regulate the expression of the H_2_S-synthesis-related gene *OsDCD1* and promote H_2_S synthesis. In addition, we found that the exogenous application of the H_2_S donor significantly alleviated the salt-sensitive phenotype of *OsNACL35-SRDX* lines under salt stress conditions, while H_2_S scavengers inhibited the salt tolerance of *OsNACL35*-*OE* lines. This suggests that H_2_S in rice plays a positive regulatory role in the resistance to salt stress, acting downstream of OsNACL35, and also supports the possibility that OsNACL35 regulates H_2_S synthesis.

H_2_S is thought to regulate the activity of target proteins through persulfidation, then exerting its biological function [68]. For example, ethylene-induced H_2_S in tomatoes was involved in osmotic-stress response by negatively regulating ethylene biosynthesis through the thiosulfylation reaction of 1-aminocyclopropane-1-carboxylate oxidase (ACO) [69]. In this study, the downstream regulatory mechanism of the signaling molecule H_2_S in rice against salt stress is not clear and needs to be further explored. Moreover, the proteomic analysis of Wei et al. revealed the potential mechanism of H_2_S protecting rice seedlings under salt stress, suggesting the possibility of H_2_S regulating biological processes, such as oxidative stress, photosynthesis, material metabolism, and cell structure, in rice [70]. Although our study found that H_2_S donor application in rice under salt stress suppressed ROS accumulation and ion leakage, suggesting that H_2_S signaling might regulate downstream oxidative stress and ion homeostasis, more time needs to be devoted to further research of the specific control mechanisms.

## 4. Materials and Methods

### 4.1. Plant Materials, Growth Conditions, and Treatments

The rice cultivars Sea Rice 86 (SR86) and Nipponbare were used in this study, in which SR86 was utilized for the analysis of expression profiling and the rice cultivar Nipponbare was used to construct the transgenic materials. For the expression profiles of *OsNACL35* in the different tissues of SR86, various tissues (root, shoot, young leaf, senescent leaf, and flower) of SR86 (120-day old) were harvested, and the tissue distribution of the *OsNACL35* transcript was detected by qRT-PCR. For the analysis of the induced expression, germinated seeds of SR86 were placed in a hydroponic medium after being immersed in water for 2 days, and then put on a light incubator (28 °C, 14/10 h light/dark cycle) for different treatments. The 14-day-old seedings of SR86 were exposed to salt stress (200 mM NaCl) and oxidative stress (H_2_O_2_) for 1 h, 3 h, 6 h, 12 h, and 24 h, and hormones (100 μM ABA, 200 μM ACC, 100 μM SA, and 100 μM IAA) as well as NaHS treatments for 3 h, respectively. The treated whole seedings were sampled at various timepoints, and three seedlings were gathered together in one sample, which were stored at −80 °C for RNA extraction.

### 4.2. RNA Isolation and qRT-PCR

Total RNA was extracted from SR86 with the M5 Total RNA Extraction Reagent Kit (Mei5 Biotechnology, Beijing, China), following the instructions for its specific operation. The first strand of cDNA was synthesized using 2 μg total RNA from SR86 with the Hiscript II Q RT SuperMix Kit (Vazyme, Nanjing, China), according to the user’s manual; then, it was stored at −20 °C. Quantitative real-time PCR (qRT-PCR) was performed using a 2 × M5 Mix PCR system (Mei5 Biotechnology, Beijing) on a Thermal Cycler Dice Real Time System III (TaKaRa, Shiga, Japan). The *OsActin* gene was used as an internal control gene, and the expression levels of genes were calculated with the 2^−^^ΔΔCT^ method. The gene-specific primers used in qRT-PCR are listed in Table 1.

### 4.3. Generation of Transgenic Lines

For generating *OsNACL35* overexpressing lines (named *OsNACL35-OE*), the coding sequences (CDS) of OsNACL35 were amplified by PCR from the rice cultivar SR86, and were cloned to the *pCAMBIA2301* vector through homologous recombination. For the *OsNACL35-SRDX* transgenic lines, the coding region of *OsNACL35* was used as a template, and the SRDX inhibition domain was added to the end of the reverse primer according to Liu et al. (2014) [14]. After that, the recombinant constructs of *pCAMBIA2301-OsNACL35* and *OsNACL35-SRDX* were transferred to the agrobacterium tumefaciens strain EHA105 via liquid nitrogen freezing and then transformed into the wild-type rice cultivar Nipponbare, as described by Mao et al. (2017) [21].

### 4.4. Subcellular Localization

To explore the localization of the OsNACL35 protein at the subcellular level, the full-length CDS sequences of *OsNACL35* were fused with the N-terminus of the green fluorescent protein (GFP) gene, driven by the cauliflower mosaic virus 35S (CaMV 35S) promoter. Then, the fusion expression gene of *35S: OsNACL35–GFP* was cloned to the expression vector *pCAMBIA2301*, producing the *pCAMBIA2301–OsNACL35–GFP* construct. After that, these constructs of 35S: GFP and 35S: OsNACL35–GFP were transformed into the agrobacterium tumefaciens strain GV3101 along with the nucleus marker Nu-mCherry and then transformed transiently into the leaves of *Nicotiana benthamiana*, respectively. Leaves were collected 48 h after infiltration for observation under a confocal laser scanning microscope (LEICA TCS SP5II, Wetzlar, Germany).

### 4.5. Transactivation Activity Assays

To further determine the transcriptional activation activity of OsNACL35, the CDS fragment of *OsNACL35* was fused with the BD domain of *pGBKT7*. The recombinant plasmids of *pGBKT7–OsNACL35*, *pGBKT7-AD*, and the empty vector *pGBKT7* were transferred into the yeast strain AHA109. The above yeast strains were plated on SD/-Trp and SD/-Trp/-His/-Ade media at 28 °C for 72 h and then subjected to the galactosidase assay.

### 4.6. Yeast One-Hybrid Assays

The coding sequence of *OsNACL35* was cloned to the *pGADT7* vector, and the *OsLEA3*(−20 to −1500 bp) and *OsDCD1* (−10 to −1600 bp) promoter fragments were constructed into the *pAbAi* vector. All vectors and empty vectors (negative control) were transformed to the yeast strain Y1HGold. The control and experimental groups were plated onto SD/-Ura-Leu and SD/-Ura-Leu + AbA (50 ng/mL and 100 ng/mL) media to observe the growth of the yeast cells at 28 °C for 72 h.

### 4.7. Physiological and Biochemical Measurements

For the chlorophyll content, the leaves of rice plants with different treatments were cut into small segments and freezed with liquid nitrogen. Chlorophyll was extracted from the above tissues in 95% ethanol, and then the absorbance was measured at 649 nm and 665 nm. Ion leakage rates were measured according to Lv et al. (2016) [71]. Briefly, the leaves of rice plants were cleaned with sterile water and afterwards immersed in deionized water. The conductivity of the exudate solution was measured before and after boiling. The Fv/Fm ratio was determined using a Hansatech m-pea fluorescence spectrometer. The content of soluble sugar was obtained with a soluble sugar content test kit (Nanjing Jiancheng), following the instructions for its specific operation. The determination of the free proline content was determined according to Alexieva et al. (2001) [72], and minor modifications were made. The determination of peroxidase (POD), superoxide dismutase (SOD), and catalase (CAT) activities was conducted as described previously (Miao et al., 2010) [73]. The hydrogen peroxide (H_2_O_2_) and superoxide anion radical (O_2_^−^) contents were determined following the descriptions by Alexieva et al. (2001) and Hou et al. (2002) [72,74], respectively.

### 4.8. Histochemical Staining

H_2_O_2_ and O_2_^−^ contents in the leaves of rice plants were determined with 3,3′-diaminobenzidine (DAB) and nitro blue tetrazolium (NBT) staining, respectively. Leaves detached from WT and transgenic lines were incubated with DAB for 40 min or NBT for 2 h. Then, the above tissues were immersed in 95% ethanol for decolorizing and, after that, transferred to 60% glycerol for imaging.

### 4.9. Measurement of Endogenous H_2_S Content

For the determination of the H_2_S content, the leaf samples were detached to measure the H_2_S content, as described by Zhang et al. (2008) with some modifications [75]. The plant tissues were homogenized with 1 mL of phosphate-buffered solution containing 0.1 M EDTA and 0.2 M AsA (pH = 7.0, 50 mM). After that, the supernatant was incubated with 1 mL of 1 M HCl to release H_2_S. The H_2_S was mixed with a 1% (*w*/*v*) zinc acetate (0.5 mL) trap for 30 min; then, 0.5 mL 5 mM N, N-dimethyl-p-phenylenediamine, and 3.5 mM H_2_SO4 were dissolved in the solution. The absorbance at 670 nm was measured for the determination of the H_2_S content.

### 4.10. Statistical Analysis

All statistical analyses were performed by a Student’s *t*-test and a two-way ANOVA among treatments. Three biological replicates for each treatment were conducted for the statistical analyses in this article. Asterisks and different letters indicate significant differences at *p* < 0.05.

## Figures and Tables

**Table 1:** Primer sequences.

Name	Forward Primer (5′-3′)	Reverse Primer (5′-3′)
*OsNACL35*	ATTGCTGTCCAATTTCAGTC	GATCATCAATAGTACATCATGAC
*OsRab16A*	CACACCACAGCAAGAGCTAAGTG	TGGTGCTCC ATCCTGCTTAAG
*OsLEA3*	CGGCAG CGTCCTCCAAC	CGGTCATCCCCAGCGTG
*OsDREB2A*	GCTGCACATCAGCACCTTCA	TCCTGC ACCTCAGGGACTAC
*OsDCD1*	GTGGATCAAGCGAGACGACA	CGCTCCCACCAATCTCTCAA
*OsERD1*	TCAAAGGGAAGACGAAGCATGG	GGGACGGAATACAACCATCTCA
*OsP5CS1*	GCTGACATGGATATGGCAAAAC	GTAAGGTCTCCATTGCATTGCA
*OsPOD*	AACGCAACCACCAAGCCG	CCTCGATCATGCCCATCTTGA
*OsCATA*	CCCCAAGGTCTCCCCTGA	AACGACTCATCACACTGGGAGAG
*OsAPX8*	ATCATCGCCAGCGGATGA	GCAGCGACGAAGGGCTC
*OsRab16A*	CACACCACAGCAAGAGCTAAGTG	TGGTGCTCC ATCCTGCTTAAG
*OsLEA3pro*	GAGTGAACAGCCGAATTCCTC	ACTTAGGATTCTCAAATTCC
*OsDCD1pro*	GATTTACATGTCAGCACCTTCA	TGACTACACCTCAGGGACTAC
